# Wounds and Diseases Involving Both Abdomen and Thorax*Abridgement of paper presented by invitation to Alabama State Medical Association during its session in Montgomery, April 13th, 1892.

**Published:** 1892-05

**Authors:** J. McFadden Gaston

**Affiliations:** Atlanta, Ga.; President Southern Surgical and Gynæcological Association; Professor of Surgery in Southern Medical College, etc.


### Southern Medical Record.

A MONTHLY JOURNAL OF MEDICINE AND SURGERY.

Vol. XXII. Atlanta, Ga., May, 1892. No. 5

### Original Articles.

### WOUNDS AND DISEASES INVOLVING BOTH ABDOMEN AND THORAX.*

BY J. M'FADDEN GASTON, OF ATLANTA, GA.,
President Southern Surgical and Gynæcological Association; Professor of Surgery in Southern Medical College, etc.

The important relations of the viscera lying above and below the diaphragm in the normal state, assume a much greater significance when they become disturbed by wounds or by pathological conditions.

Certain parts of the vital organism belong exclusively to the thoracic or to the abdominal region, while other structures extend through the partition, so as to occupy both of these divisions. To the latter class belong the aorta, the vena cava, the great sympathetic nerve, the lymphatic system and their respective ramifications. The alimentary mechanism is intimately connected with both compartments, while all the elaborating processes are carried on in the lower division. All the movements and sensations of the body depend upon the supply of nerve power by the medulla spinalis, which, included in its bony casing, sends out its lines of communication as it descends

*Abridgement of paper presented by invitation to Alabama State Medical Association during its session in Montgomery, April 13th, 1892.

on the posterior portion of the thorax and the abdomen. With these vascular, lymphatic and nervous communications between the organs included in the upper and lower cavities of the body, we are prepared to understand how the troubles of each may be propagated to the other. While the principle of sympathy between the different structures is a recognized pathological condition, it is explained on the same basis of effects which characterizes reflex disturbances, and must be accounted for through the interlacing of the nerve filaments.

The ordinary sequence of a local disorder, whether traumatic or idiopathic, is not generally distributed to all the surrounding structures, but in certain directions and by fixed channels to a definite class of tissues under the influence of pathological laws.

The revolution is notable through which thoracic and abdominal surgery has passed in the last quarter of a century. The favorable results of thoracotomy and laparotomy demonstrate a recuperative power on the part of the pleura and peritoneum which is well illustrated by a case of extensive wound reported by Brokaw, of St. Louis.

A typical case of thoraco-abdominal wound occurred under my observation in the early part of 1891, in the person of a young negro man. While engaged in a fight he was stabbed in the left side, the blade of the knife entering above the tenth rib on a line descending perpendicularly from the nipple. It passed inward and downward through the pleural cavity, penetrating the diaphragm and invaded the abdominal cavity. When I was called to him at Providence Infirmary, some hours after the injury was received, it was ascertained that he had vomited repeatedly from the effects of beer which he had been drinking. He lay in a semi-conscious state when left alone, but became agitated and violent when disturbed, and was therefore given a hypodermic of morphine, grain 1-4, atropia, grain 1-150, to secure composure for the surgical operation.

Upon examination, I found a hernial protrusion of the omentum about the size of a hen egg outside of the cutaneous opening. Pressing steadily with my fingers around and above this mass, it was gradually carried back through the external wound. But upon pressing my index finger within and following up the tissue of the hernia, it was passed through an incision through the diaphragm and lodged in the abdominal cavity. As the opening in the diaphragm lay entirely below the opening in the skin, it was found practicable to have pressure made effectually by the fingers of an assistant placed below the tenth rib, so as to occlude the incision in the diaphragm, and thus prevent the recurrence of the protrusion. I then proceeded to unite the edges of the wound by suture, passing the needle deeply through the margins of the divided intercostal tissues, thus bringing about a complete coaptation of the wounded thoracic wall.

With a view to compress the abdominal viscera from above downwards, a firm roll of cotton was secured over the wound and the lower ribs by a bandage carried around the waist. The patient was kept under the influence of an opiate in the recumbent position, without taking any fluid into the stomach, and there was no return of the vomiting, which had evidently forced the omentum upwards through the opening in the diaphragm and the chest wall. No unfavorable symptoms followed the injury, and upon removing the stitches at the end of a week, the external wound had healed completely by first intention.

The most important class of injuries to the abdominal and thoracic viscera are those inflicted by fire arms. The medico-legal aspect of gunshot wounds arrests attention especially, and the difficulty of determining upon the course of a ball in either of these cavities when there is only a point of entrance brought under the observation of the surgeon is generally recognized.

The practical bearing of a case, which was examined in conjunction with several colleagues, warrants a report of the facts presented at the outset and subsequently.

The patient received a pistol-shot in the epigastric region, and no clue was obtained as to the course of the ball until two days afterwards when soreness directed attention to the right side of the chest. Upon palpation the ball was found lodged between the seventh and eighth ribs, and hence must have passed diagonally inward and upward. It must have traversed the liver and the lower part of the pleural cavity, with perhaps injury of the lung. There was a marked tendency to collapse about twenty-four hours after the injury was received, which was attributed to hemorrhage into the right pleura. He rallied under the use of stimulants hypodermically applied, but died at the close of three days and no post-mortem examination was made.

An instructive case of gunshot wound penetrating the upper portion of the chest, on the right side, and passing downward diagonally across to the left side of the abdomen, came under my observation a few years ago, in this city. I saw this patient in consultation about a month after the injury was received and then diagnosed empyæma of the right pleural cavity, with the recommendation of an operation. But, on the following day, another colleague with a good surgical standing in the profession, joined in the consultation and gave an opinion in favor of waiting for further developments. My conviction was unchanged in regard to the urgency for evacuating the collection, but the attendant acquiesced in adopting the course of masterly inactivity in the case, and the patient succumbed in less than a week afterwards.

Being invited to participate in the post-mortem examination, it was verified that the right pleural cavity was filled with pus to such an extent as to push the mediastinum beyond the median line and to cause a depression of the diaphragm. The ball had traversed the liver and was found encysted in the omentum, with but slight indication of inflammation in the peritoneum or other abdominal viscera.

A counterpart of this last case has come under my observation recently in a gunshot wound entering the left side of the chest and ranging diagonally backward into the abdomen.

This was a white man, A. C, thirty-three years old, who was shot with a Smith & Wesson pistol of 38 calibre by a man at his left side. I saw him about fifteen minutes after the shooting and found that the ball had passed through a thick coat, vest, shirt and undershirt, penetrating the chest wall between the sixth and seventh ribs on a line with the anterior auxillary fold, and about an inch below a transverse line from the left nipple. It was out of my power to determine precisely what course the ball had taken, and upon a careful examination of the opposite side of the thorax by palpation, the presence of the ball could not be detected.

The patient labored under the influence of shock to a limited extent with a weak and frequent pulse, but there was no hemoptysis or dullness upon percussion over the left side, and the respiratory murmur was very evident upon auscultation over the front of the left lung. It was hence inferred that the ball had not penetrated the lung. About twenty minutes after the accident a hypodermic injection of morphine gr. 1-4, atropia gr. 1-150 was used and repeated in half an hour.

Upon learning two hours afterwards that he had vomited blood, a combination of ergot, digitalis and elixir of opium was given. Realizing that the stomach was perforated, I suggested next morning a laparotomy, but my colleague thought it best to leave the case to nature, and the patient was left in his charge.

As this kind of surgical conservatism was not calculated to avail for the relief of perforation of the stomach, it was not a matter of surprise that the case terminated fatally at the end of the third day from the night of the injury.

An autopsy made by the county physician, Dr. E. Griffin, under the direction of the coroner, revealed that the ball had passed through the thoracic wall, traversing the lower portion of the left pleural cavity just beneath the pericardium, thence perforating the diaphragm and the walls of the stomach, with those of the transverse colon. The conical ball was found lodged in the muscular tissues on the left side of the vertebral column, nearly opposite the seventh dorsal vertebra. There was but little blood in the pleura, but much in the peritoneum.

When the anatomy of all the parts involved is recognized, it will be understood that the ball passed nearly at a right-angle with the axis of the body and came from a pistol held on a line with the external wound, by a person occupying a position obliquely on the left of the victim, as described by an eye-witness.

The matter of practical moment in this case is the probable outcome of a laparotomy after the wound of the stomach was recognized by the vomiting of blood. In view of the fact revealed by the autopsy, that the colon and stomach were perforated, and that no other serious wounds existed, it appears that closing the openings might have averted death.

An interesting case, in which an injury over the sternum extended to the abdomen, has been treated in consultation in the person of a vigorous white man, with a fatal result.

A contused wound, without laceration of the skin or fracture of the bone, caused considerable pain in the chest at the outset, followed by febrile disturbances, and was ultimately complicated with cramp of the bowels and distension of the abdomen. There was marked thirst and vomiting from taking large quantities of fluids into the stomach. The intestinal torpor only yielded to frequent doses of calomel, followed by castor oil and spirits of turpentine.

While there were indications of traumatic pneumonia as a direct result of the injury, the transmission of the inflammatory process to the abdominal viscera induced peritonitis of a grave character. So far as could be learned from the history of the contusion, there was no violence immediately to the walls of the abdomen, and hence it was inferred that, by a metastatic process inflammation was propagated to the peritoneum. Notwithstanding the employment of vigorous measures to combat the thoraco-abdominal complication, it ended fatally in the course of a week.

In illustration of the effects of pathological conditions implicating the abdomen and thorax, I recall an interesting case connected with gall-stones, which was under my care some years ago.

After long continued hepatic derangement, with evacuations of gall-stones by the bowels from time to time, there eventually developed a pulmonary disease with a very peculiar expectoration. It did not present the characteristic appearance of mucus or pus, but had a grumous and sanious character, with a most offensive odor, such as has never been encountered in any other case. This very remarkable stench permeated the apartments, so as to make it extremely unpleasant to remain in any part of the house, and there was a constant renewal of it from frequent expectoration of the dark brown matter by coughing. The patient had been for several weeks in a very feeble condition, and only survived this pulmonary complication a few days.

An autopsy revealed several very interesting pathological complications.

An ulcerated communication between the abdomen and the thorax was the feature of the case bearing most directly upon the subject of this paper, and under peculiar conditions.

The precursory hepatic troubles in this case were so marked, that the subsequent developments were properly interpreted prior to the post-mortem examination and yet I was not prepared to expect such a complete exclusion of the pleural cavity by the adhesions of the lung with the diaphragm. A tract of escape for the morbid products from the liver through the bronchial ramifications was opened up by the ulceration process, and had not the powers of the patient been already exhausted, it is infused that the drain thus established might have afforded relief.

The following report has been received from Dr. D. A. K. Steele, of Chicago, in reply to my request for some notes in cases of liver abscess perforating the diaphragm:

"I beg to say that in 1874, I had under my care in the Cook Co. Hospital, a patient, male, aged thirty years, who was admitted to a medical ward, suffering from a severe cough, expectoration of large quantities of thick greenish yellow purulent sputum, occasionally tinged with blood. There was great emaciation, hectic, temperature and the physical evidences of an empyæma, discharging through a bronchus. He was treated for empyæma for about ten days, when he died, and, at the post-mortem examination we found an abscess of large size in the right lobe of the liver that had ulcerated through the diaphragm into the right pleural cavity and was discharging through a large bronchial tube. It was an instructive case, as the liver abscess origin of the empyæma had not been suspected."

From statistics of abscesses of the liver, published by a famous Mexican physician, Jimenez, it appeared that in 15 cases an opening occurred before incision into the bronchi. In four cases, after incision, the discharges passed into the bronchi. In one case the opening was into the stomach, in one case into the pericardium, and in two cases into the pleuræ.

Thus, it is found that the communication between the abdominal and thoracic cavities, by ulceration through the diaphragm, is more frequent than might have been expected the form paucity of recorded data in this country.

The difficulties attending a correct understanding of the complications growing out of thoraco-abdominal wounds and diseases are such as to demand full investigation of this class of cases by the profession.

The anatomical relations of the thoracic and abdominal viscera, separated by the concavo-convex muscular partition of the diaphragm, renders a diagnosis of complex injuries highly important.

I have ventured upon this comparatively unexplored field of observation, with the hope of arousing an interest in this branch of surgery; and I trust that the points presented may elicit profitable discussion from those who may have experience in treating these complications.

